# Serum apolipoprotein A-IV levels are associated with flow-mediated dilation in patients with type 2 diabetes mellitus

**DOI:** 10.1186/s12872-022-02898-x

**Published:** 2022-10-25

**Authors:** Le-Ying Li, Shuai Chen, Yi-Xuan Wang, Ri Ji, Feng-Hua Ding, Xiao-Qun Wang, Qiu-Jing Chen, Lin Lu, Yang Dai

**Affiliations:** 1 Department of Cardiovascular Medicine, Ruijin Hospital, Shanghai Jiao Tong University School of Medicine, 197 RuiJin Road II, Shanghai, 200025 People’s Republic of China; 2 Department of Ultrasound, Ruijin Hospital, Shanghai Jiao Tong University School of Medicine, Shanghai, People’s Republic of China; 3 Institute of Cardiovascular Diseases, Shanghai Jiao Tong University School of Medicine, Shanghai, People’s Republic of China

**Keywords:** Flow-mediated dilation, Endothelial function, Apolipoprotein A-IV, Type 2 diabetes mellitus

## Abstract

**Background:**

Endothelial dysfunction is common in diabetes. Apolipoprotein (apo) A-IV functions to antagonize inflammation and oxidative stress. The present study aimed to investigate the relationship between flow-mediated dilation (FMD) and serum apoA-IV level in type 2 diabetes mellitus (T2DM) patients.

**Methods:**

A total of 84 T2DM patients with chest discomfort were enrolled in this study. Their baseline characteristics and clinical parameters were documented. Endothelial function of the participants was evaluated by examining FMD of brachial artery. The severity of coronary atherosclerosis was determined by quantitative coronary angiography. Serum apoA-IV levels were measured by ELISA.

**Results:**

These diabetic patients were dichotomized into low FMD (*n* = 42) and high FMD (*n* = 42) groups. Serum apoA-IV levels were significantly higher in high FMD group than in low FMD group (29.96 ± 13.17 vs 17.69 ± 9.16 mg/dL, *P* < 0.001). Moreover, the patients were also categorized into three apoA-IV tertile groups. FMD was significantly different across three apoA-IV tertiles (*P* < 0.001). Serum apoA-IV levels were positively correlated to FMD (*r* = 0.469, *P* < 0.001). Logistic regression analysis was performed to determine risk factors for low FMD. apoA-IV levels together with the risk factor hsCRP remained significantly to be independent determinants of low FMD (*P* < 0.01). Linear regression analysis was performed, and apoA-IV levels together with total-to-HDL cholesterol ratio were independently correlated with FMD (*P* < 0.01).

**Conclusions:**

Serum apoA-IV levels are associated with FMD, suggesting that apoA-IV protects endothelial function in patients with T2DM.

## Introduction

Diabetes is a crucial risk factor for atherosclerosis. Diabetes induces formation of advanced glycated end-product formation, oxidative stress and chronic inflammation, leading to arterial endothelial dysfunction and development of atherosclerotic cardiovascular diseases [1, 2]. Impairment of endothelial function precedes structural changes of atherosclerosis and plays a central role throughout the whole process of atherosclerosis [3, 4]. Test of endothelial function allows ascertainment of arterial physiology and pathology status. Flow-mediated dilation (FMD) is a non-invasive tool for examining peripheral artery endothelium-dependent dilation with high-resolution ultrasonography. The dilation is largely nitric oxide (NO)-mediated process in response to sudden increase in blood flow or shear stress [5–7]. FMD relates to endothelial function and independently predicts cardiovascular events [8].

ApoA-IV is a glycoprotein synthesized mainly by the small intestine [9]. The majority of circulating apoA-IV is lipid-free or associated with chylomicrons, with a minor portion related to HDL [10]. ApoA-IV has been proved to be atheroprotective due to positive role in reverse cholesterol transport [11, 12], intestinal lipid absorption [13], glucose homeostasis, insulin secretion [14] and the properties of anti-oxidation and anti-inflammation [15, 16]. However, the relation of apoA-IV and endothelial function remains unclear, especially in diabetic milieu of which vascular endothelium is probably impaired.

Thus, the present study investigated the relationship between vascular endothelial function and serum apoA-IV levels in patients with T2DM. The endothelial function of diabetic patients was evaluated by examining FMD. Serum apoA-IV levels were determined by ELISA.

## Methods

The study followed the principles of outlined in the Declaration of Helsinki, and written informed consent was obtained from all participants.

### Study population and samples

**Fig. 1:**
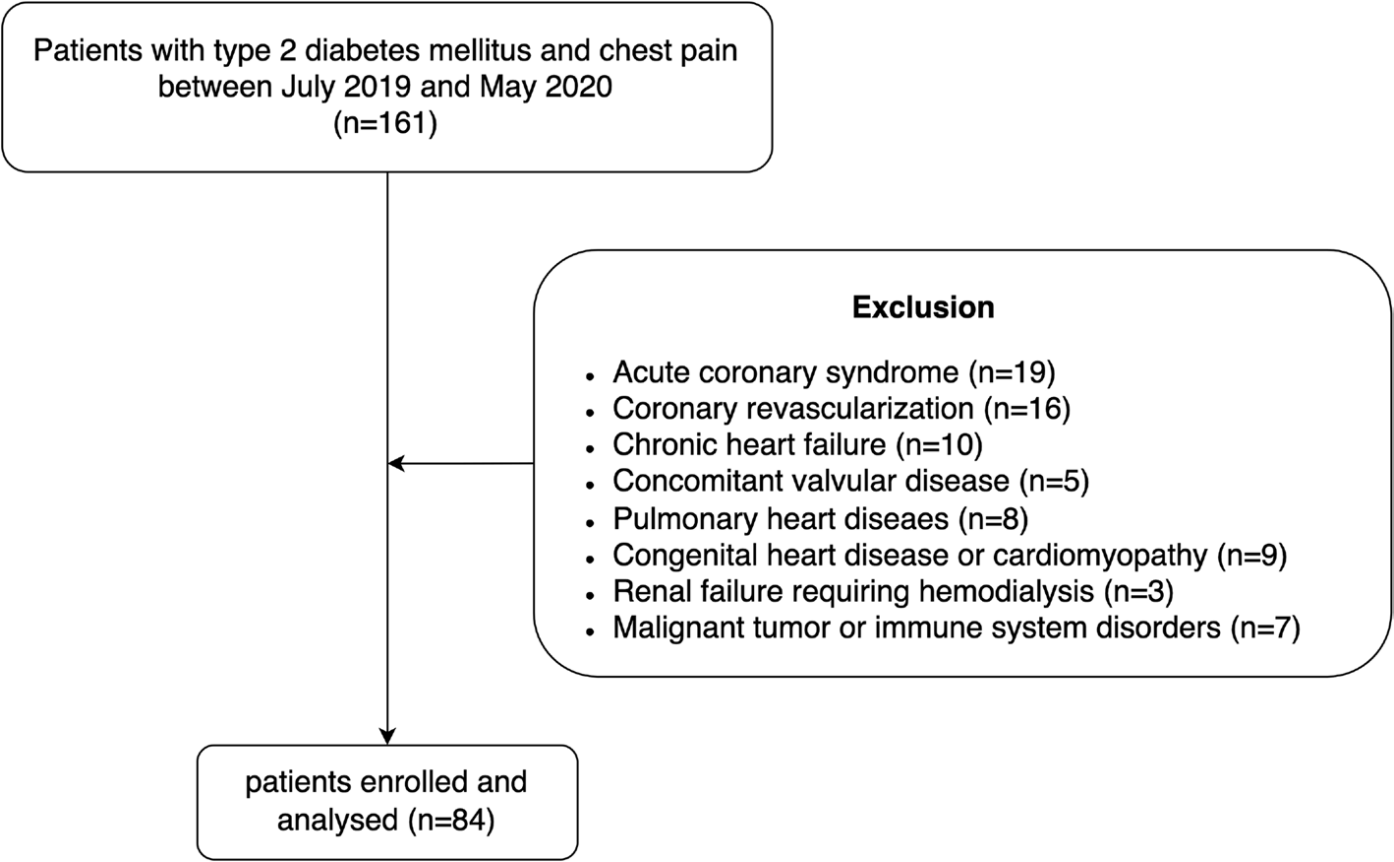
Flowchart of patient enrollment

A total of 161 T2DM patients with paroxysmal chest discomfort undergoing coronary angiography from July 2019 to May 2020 for the diagnosis of coronary artery disease were enrolled in the present study. For the purpose of research, we excluded patients with following diseases including acute coronary syndrome (*n* = 19), history of coronary revascularization (*n* = 16), chronic heart failure (*n* = 10), concomitant valvular disease (*n* = 5), pulmonary heart disease (*n* = 8), congenital heart disease or cardiomyopathy (*n* = 9), renal failure requiring hemodialysis (*n* = 3) and malignant tumor or immune system disorders (*n* = 7). In the end, 84 patients were enrolled (Fig. 1).

Serum samples were obtained from patients after 12 h fasting. These samples were stored at − 80 °C until analysis.

### Coronary angiography

Coronary angiography was performed through the femoral or radial approach, using the Cardiovascular Measurement System version 3.0 software (Terra, GE, USA). All angiograms were reviewed by two experienced interventional cardiologists, both of whom were blinded to the study protocol and clinical data. A judgment was made by a third cardiologist if these two doctors had disagreement on lesion severity. The analysis of coronary lesion was performed as described previously [17].

### Flow-mediated dilation

FMD on brachial artery of right upper arm was evaluated by an experienced ultrasound doctor, who was blinded to study design and clinical data of the participants, by using a high-resolution ultrasound machine with a 10‐MHz linear array probe and the GE Vivid 7 Imaging System following the recommended protocol [18, 19]. Briefly, the process was to measure the brachial artery diameter by recording the distance between the proximal and distal end of the intimal at the end of diastole (gated by R wave on electrocardiogram). The blood pressure cuff was banded distal to the imaged artery and held inflated on patients’ right upper arm for 5 min at 200 mmHg with the help of an assistant. FMD measurements were taken continuously from deflation for no less than 3 min. Baselines were recorded before inflation at the same artery segment under quiet and temperature-controlled circumstances for at least 1 min. The final FMD data is calculated as ratio of maximum brachial artery dilation to the baseline value.

### ApoA-IV measurement

Serum apoA-IV levels were measured with HUMAN APOLIPOPROTEIN IV (ApoA4) ELISA kit (SK00401-01) according to the instructions provided by the manufacturer (AVISCERA BIOSCIENCE, INC.). ApoA-IV levels were determined by comparing OD values on 450 nm with a standard curve of gradient dilution of human recombinant apoA-IV protein. ApoA-IV levels were presented as mg/dL.

### Statistical analyses

Continuous variables are presented as mean ± standard deviation (SD), and categorical data are summarized as frequency (percentage). For continuous variables, normal distribution was evaluated with the Kolmogorov–Smirnov test. Differences among groups were analyzed by one-way analysis of variance (ANOVA) and significance of trend was analyzed by linear ANOVA. For categorical clinical variables, differences between groups were evaluated by the linear chi-square test or Fisher’s exact test. Correlation between factors was analyzed by Pearson correlation test. Multivariable logistic regression models were performed to assess the independent determinants of FMD without (Model 1) and with apoA-IV (Model 2). Multivariable linear regression was performed to assess independent factors correlated with FMD. Overall significance level (2-tailed) of 0.05 was set as criterion. All statistical analyses and figures were performed with IBM SPSS Version 26 for Mac (IBM SPSS Inc, Chicago, IL, USA), Prism 9 for macOS (1994—2021 GraphPad Software, LLC) and Adobe Illustrator 23.1.1.

## Results

### Serum apoA-IV levels are higher in diabetic patients with high FMD, and FMD is significant different across three apoA-IV tertiles

The diabetic patients (*n* = 84) were dichotomized into low FMD (*n* = 42, 1.26% ~ 5.88%) and high FMD (*n* = 42, 5.88% ~ 13.58%) groups. The baseline characteristics and clinical parameters of these two FMD groups were detailed in Table 1A. Elder age and high apoB level were observed in low FMD group as compared with high FMD group. Notably, serum apoA-IV levels were significantly higher in high FMD group than in low FMD group (29.96 ± 13.17 vs. 17.69 ± 9.16, *P* < 0.001).

**Table 1:** Diabetic patient characteristics

**A) Diabetic patient characteristics sorted by FMD**				
	Low FMD (*n* = 42)	High FMD (*n* = 42)	*P* value	
Male, n (%)	30 (71.43)	32 (76.19)	0.620	
Age, years	66.45 ± 7.97	64.04 ± 8.455	**0.008**	
BMI, kg/m^2^	27.55 ± 3.29	27.55 ± 2.96	0.492	
Smoke, n (%)	12 (28.57)	19 (45.24)	0.113	
Hypertension, n (%)	31 (73.80)	32 (76.19)	0.801	
SBP, mmHg	138.26 ± 18.3	137.69 ± 18.8	0.782	
DBP, mmHg	74.33 ± 10.52	74.94 ± 11.39	0.628	
FBG, mmol/L	6.81 ± 3.04	6.63 ± 2.47	0.516	
HbA1c, %	7.64 ± 1.17	7.53 ± 1.26	0.428	
HOMA-IR	4.94 ± 4.21	3.78 ± 2.44	0.126	
DM duration, years	9.79 ± 7.94	6.02 ± 4.54	**0.009**	
Dyslipidemia, n (%)	22 (52.38)	24 (57.14)	0.661	
Triglyceride, mmol/L	1.60 ± 1.02	1.63 ± 1.40	0.861	
Total cholesterol, mmol/L	3.95 ± 1.04	3.88 ± 0.99	0.498	
HDL-C, mmol/L	1.14 ± 0.26	1.15 ± 0.26	0.630	
LDL-C, mmol/L	2.21 ± 0.65	2.24 ± 0.72	0.675	
ApoA, g/L	1.20 ± 0.22	1.20 ± 0.20	0.775	
ApoB, g/L	0.77 ± 0.28	0.70 ± 0.16	**0.024**	
Lp(a), g/L	0.26 ± 0.29	0.28 ± 0.30	0.484	
BUN, mmol/L	6.63 ± 2.94	6.20 ± 2.32	0.087	
Serum creatinine, μmol/L	80.86 ± 21.85	80.41 ± 18.52	0.826	
eGFR, ml·min^−1^·1.73 m^−2^	80.7 ± 19.00	83.36 ± 16.46	0.140	
UA, μmol/L	341.98 ± 111.29	349.87 ± 96.79	0.458	
hsCRP, mg/L	6.81 ± 20.5	5.36 ± 16.55	0.427	
Gensini score	74.95 ± 64.55	34.06 ± 45.96	**0.001**	
SYNTAX score	21.56 ± 16.46	11.10 ± 13.51	**0.002**	
**ApoA-IV, mg/dL**	17.69 ± 9.16	29.96 ± 13.17	** < 0.001**	
Medication, n (%)				
ACE inhibitor	22 (52.38)	24 (57.14)	0.661	
β-blocker	32 (76.19)	26 (61.90)	0.157	
Calcium channel blocker	14 (33.33)	12 (28.57)	0.637	
Statins	36 (85.71)	40 (95.24)	0.137	
Antiplatelet	23 (54.76)	18 (42.86)	0.275	
Metformin	23 (54.76)	23 (54.76)	1	
Sulfonylureas	9 (21.43)	7 (16.67)	0.782	
DPP-4 inhibitors	1 (2.38)	1 (2.38)	1	
SGLT2 inhibitors	4 (9.52)	0	0.116	
Meglitinides	3 (10.71)	6 (21.43)	0.480	
Thiazolidinediones	2 (4.76)	0	0.494	
α-glucosidase inhibitors	12 (28.57)	12 (28.57)	1	
Insulin	15 (35.71)	11 (26.19)	0.345	
**B) Diabetic patient characteristics sorted by ApoA-IV tertiles**				
	ApoA4 Tertile1 (*n* = 28)	ApoA4 Tertile2 (*n* = 28)	ApoA4 Tertile3 (*n* = 28)	*P* for trend
Male, n (%)	20 (71.43)	21 (75.00)	21 (75.00)	0.763
Age, years	66.07 ± 7.69	65.46 ± 7.48	60.57 ± 9.26	**0.014**
BMI, kg/m^2^	27.16 ± 3.22	27.78 ± 2.43	27.71 ± 3.23	0.492
Smoke, n (%)	8 (28.57)	10 (35.71)	13 (46.43)	0.169
Hypertension, n (%)	21 (75.00)	19 (67.86)	24 (85.71)	0.349
SBP, mmHg	137.32 ± 15.86	139.93 ± 22.56	135.82 ± 17.82	0.768
DBP, mmHg	75.00 ± 11.38	71.96 ± 11.69	77.86 ± 10.72	0.346
FBG, mmol/L	7.82 ± 3.56	6.10 ± 1.33	5.98 ± 1.44	**0.004**
HbA1c, %	7.94 ± 1.57	7.11 ± 0.80	7.54 ± 1.18	0.225
HOMA-IR	4.64 ± 2.41	3.44 ± 1.75	4.99 ± 5.16	0.704
DM duration, years	9.14 ± 7.70	7.64 ± 6.49	6.93 ± 5.83	0.456
Dyslipidemia, n (%)	16 (57.14)	14 (50.00)	16 (57.14)	1.000
Triglyceride, mmol/L	1.36 ± 0.67	1.49 ± 0.88	2.04 ± 2.14	0.073
Total cholesterol, mmol/L	4.08 ± 1.10	3.63 ± 1.00	3.93 ± 0.82	0.551
HDL-C, mmol/L	1.16 ± 0.28	1.16 ± 0.26	1.14 ± 0.24	0.677
LDL-C, mmol/L	2.39 ± 0.74	2.00 ± 0.70	2.34 ± 0.68	0.817
ApoA, g/L	1.20 ± 0.21	1.21 ± 0.18	1.20 ± 0.21	0.990
ApoB, g/L	0.80 ± 0.40	0.70 ± 0.20	0.80 ± 0.18	0.919
Lp(a), g/L	0.26 ± 0.33	0.30 ± 0.29	0.29 ± 0.28	0.703
BUN, mmol/L	6.13 ± 1.61	6.58 ± 3.32	5.88 ± 1.61	0.680
Serum creatinine, μmol/L	79.43 ± 15.45	82.00 ± 22.43	79.80 ± 17.56	0.941
eGFR, ml·min^−1^·1.73 m^−2^	82.73 ± 14.40	81.25 ± 19.00	86.10 ± 15.85	0.448
UA, μmol/L	337.18 ± 79.00	357.43 ± 110.06	355.00 ± 100.43	0.496
hsCRP, mg/L	6.29 ± 21.28	4.44 ± 14.10	5.35 ± 13.73	0.834
Gensini score	62.77 ± 56.75	61.68 ± 70.13	39.07 ± 48.21	0.137
SYNTAX score	17.27 ± 16.20	18.04 ± 17.50	13.68 ± 13.92	0.402
**FMD, %**	5.00 ± 1.81	6.50 ± 2.63	7.22 ± 1.70	** < 0.001**
Medication, n (%)				
ACE inhibitor	13 (46.43)	16 (57.14)	17 (60.71)	0.286
β-blocker	19 (67.86)	19 (67.86)	20 (71.43)	0.774
Calcium channel blocker	6 (21.43)	12 (42.86)	8 (28.57)	0.566
Statins	25 (89.29)	24 (85.71)	27 (96.43)	0.365
Antiplatelet	13 (46.43)	10 (35.71)	18 (64.29)	0.184
Metformin	19 (67.86)	11 (39.29)	16 (57.14)	0.095
Sulfonylureas	6 (21.43)	6 (21.43)	4 (14.29)	0.734
DPP-4 inhibitors	0	1 (3.57)	1 (3.57)	/
SGLT2 inhibitors	2 (7.14)	1 (3.57)	1 (3.57)	/
Meglitinides	4 (14.29)	2 (7.14)	3 (10.71)	/
Thiazolidinediones	2 (7.14)	0	0	/
α-glucosidase inhibitors	9 (32.14)	8 (28.57)	7 (25.00)	0.839
Insulin	11 (39.29)	10 (35.71)	5 (17.86)	0.178

Data are mean ± SD or number (%)

*Abbreviations*: *FMD* Flow-mediated dilation, *BMI* Body mass index, *SBP* Systolic blood pressure, *DBP* Diastolic blood pressure, *FBG* Fasting blood glucose, *HbA1c* Glycosylated hemoglobin A1c, *HOMA-IR* Homeostatic model assessment for insulin resistance, *LDL-C* Low-density lipoprotein cholesterol, *HDL-C* High-density lipoprotein cholesterol, *BUN* Blood urea nitrogen, *UA* Uric acid, *eGFR* estimated glomerular filtration rate, *hsCRP* high-sensitivity C reactive protein, *DPP-4* Dipeptidyl peptidase-4, *SGLT2* Sodium-glucose co-transporter 2

**Fig. 2:**
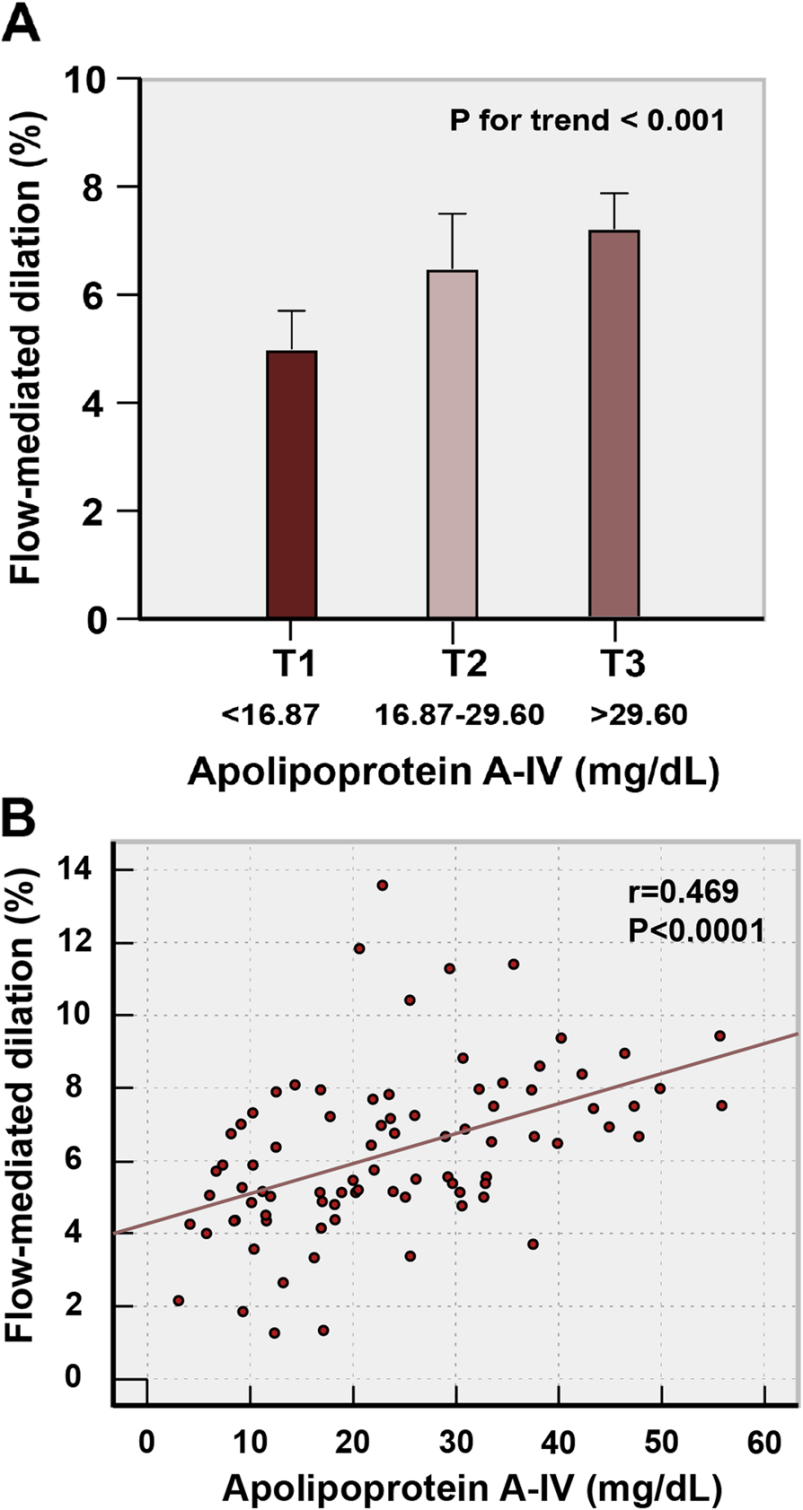
FMD and apoA-IV. (**A**) FMD distribution in apoA-IV tertiles in diabetic patients; (**B**) Correlation between FMD and apoA-IV in diabetic patients

These diabetic patients (*n* = 84) were also categorized into three apoA-IV tertile groups (Table 1B), with the range of three ApoA-IV tertiles as follow, tertile 1, < 16.87 mg/dL; tertile 2, 16.87–29.60 mg/dL and tertile 3, > 29.60 mg/dL. Significant difference regarding age (*P* < 0.05) and fasting blood glucose (*P* < 0.01) was observed among the three tertile groups. Importantly, FMD (*P* < 0.001) was significantly different across the three apoA-IV tertiles (Fig. 2A). Moreover, Serum apoA-IV levels were positively correlated to FMD in all the diabetic patients (*r* = 0.469, *P* < 0.001) (Fig. 2B). As for the severity of CAD, Gensini score and SYNTAX score were both observed declining from apoA-IV tertile 1 to tertile 3, though the trends weren’t statistically significant.

### Decreased ApoA-IV level is an independent determinant of low FMD in patients with T2DM

We performed logistic regression analyses to determine risk factors for low FMD. In model 1 (Table 2), major risk factors in Table 1 including male, age, BMI, hypertension, smoking, eGFR, hsCRP, HbA1c, HOMA-IR, DM duration, and total-to-HDL cholesterol ratio were included. The result showed that hsCRP (OR = 1.469, 95%CI 0.993–2.171, *P* < 0.05) was significantly associated with low FMD. In model 2, apoA-IV was included together with the other risk factors in model 1. Decreased ApoA-IV (OR = 0.906, 95%CI 0.856–0.959, *P* < 0.01) and hsCRP (OR = 1.621, 95%CI 1.018–2.580, *P* < 0.05) remained significantly to be independent determinants of low FMD, with calibrations of both models as follows, *P* = 0.872 for Model 1 and *P* = 0.930 for Model 2 in Hosmer–Lemeshow test. The addition of apoA-IV in Model 2 significantly improved predictive performance with an increase of Nagelkerke *R*^2^ by 17.8%. In multiple linear regression analysis, apoA-IV (β = 0.407, *P* < 0.001) and total-to-HDL cholesterol ratio (β = -0.258, *P* < 0.01) were independently correlated with FMD (Table 3).

**Table 2:** Logistic regression analyses for low FMD in diabetic patients

	Variables	OR (95% CI)	*P* value
Model 1	Male	0.819 (0.226 ~ 2.966)	0.761
Nagelkerke *R*^2^ = 0.304	Age	1.076 (0.993 ~ 1.167)	0.073
Hosmer–Lemeshow test:	BMI	0.963 (0.800 ~ 1.161)	0.695
*P* = 0.872	Hypertension	0.777 (0.225 ~ 2.681)	0.690
	Smoke	0.561 (0.180 ~ 1.748)	0.319
	eGFR	0.999 (0.959 ~ 1.040)	0.956
	Log hsCRP	1.469 (0.993 ~ 2.171)	**0.049**
	HbA1c	0.978 (0.646 ~ 1.480)	0.915
	HOMA-IR	1.125 (0.914 ~ 1.385)	0.265
	DM duration	1.079 (0.992 ~ 1.174)	0.077
	Total-to-HDL cholesterol ratio	1.278 (0.728 ~ 2.274)	0.386
Model 2	Male	0.705 (0.151 ~ 3.283)	0.656
Nagelkerke *R*2 = 0.482	Age	1.052 (0.961 ~ 1.152)	0.271
Hosmer–Lemeshow test:	BMI	1.018 (0.821 ~ 1.275)	0.877
*P* = 0.930	Hypertension	1.179 (0.304 ~ 4.579)	0.812
	Smoke	0.911 (0.244 ~ 3.399)	0.890
	eGFR	0.995 (0.951 ~ 1.040)	0.812
	Log hsCRP	1.621 (1.018 ~ 2.580)	**0.042**
	HbA1c	0.824 (0.502 ~ 1.351)	0.442
	HOMA-IR	1.112 (0.907 ~ 1.364)	0.305
	DM duration	1.091 (0.987 ~ 1.205)	0.088
	Total-to-HDL cholesterol ratio	1.346 (0.680 ~ 2.663)	0.394
	ApoA-IV	0.906 (0.855 ~ 0.959)	**0.001**

Model 1, adjusted for conventional cardiovascular factors

Model 2, adjusted for the factors included in Model 1 with the addition of apoA-IV

*BMI* Body mass index, *eGFR* estimated glomerular filtration rate, *hsCRP* high sensitive C reactive protein, *HbA1c* glycosylated hemoglobin A1c, *HOMA-IR* Homeostatic model assessment for insulin resistance, *HDL* High-density lipoprotein, *FMD* Flow-mediated dilation

**Table 3:** Linear regression analyses for FMD in diabetic patients

Variables	Unstandardized coefficients		Standardized coefficients (β)	*P* value	95% CI
	B	SE			
Male	0.149	0.558	0.029	0.791	-0.964 ~ 1.261
Age	-0.034	0.033	-0.128	0.303	-0.100 ~ 0.032
BMI	0.005	0.081	0.006	0.955	-0.156 ~ 0.165
Hypertension	-0.441	0.519	-0.083	0.398	-1.475 ~ 0.593
Smoke	0.042	0.488	0.009	0.931	-0.930 ~ 1.015
eGFR	0.001	0.017	0.006	0.961	-0.033 ~ 0.035
Log hsCRP	-0.279	0.159	-0.176	0.083	-0.596 ~ 0.038
HbA1c	-0.020	0.181	-0.011	0.914	-0.381 ~ 0.342
HOMA-IR	-0.044	0.065	-0.068	0.499	-0.173 ~ 0.085
DM duration	-0.059	0.034	-0.174	0.092	-0.127 ~ 0.010
Total-to-HDL cholesterol ratio	-0.577	0.215	-0.258	**0.009**	-1.007 ~ -0.148
ApoA-IV	0.072	0.018	0.407	** < 0.001**	0.037 ~ 0.107

*BMI* Body mass index, *eGFR* Estimated glomerular filtration rate, *hsCRP* High sensitive C reactive protein, *HbA1c* glycosylated hemoglobin A1c, *HOMA-IR* Homeostatic model assessment for insulin resistance, *HDL* High-density lipoprotein, *FMD* flow-mediated dilation

**Fig. 3:**
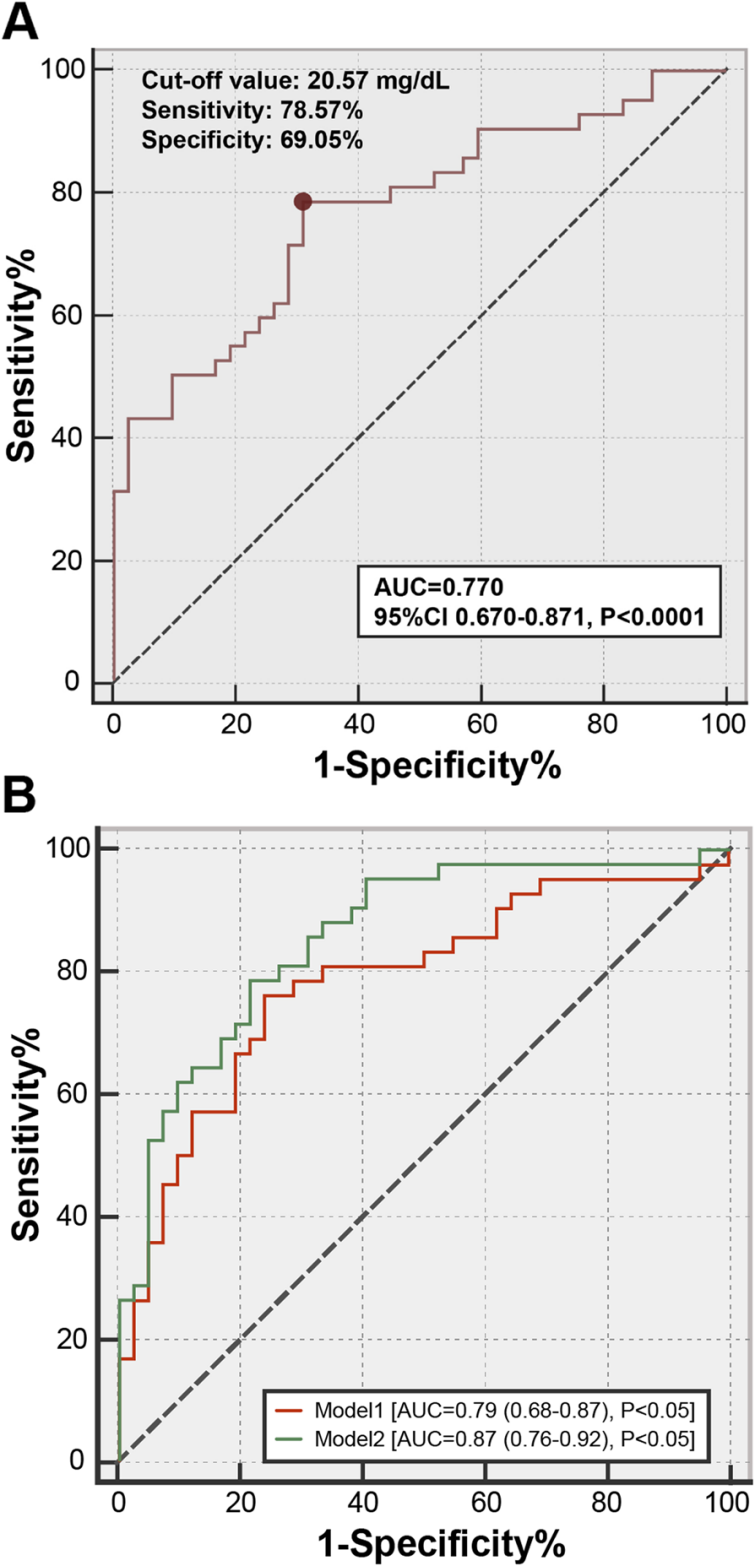
ROC curves. (**A**) ROC of apoA-IV for detecting low FMD in diabetic patients; (**B**) ROC of Model 1 and Model 2 for detecting low FMD in diabetic patients

In addition, the cut-off point of apoA-IV in predicting low FMD was 20.57 mg/dL, with sensitivity 78.57% and specificity 69.05% (Fig. 3A). ROC curves for both models (Fig. 3B) showed that the addition of apoA-IV in Model 2 effectively elevated AUC (AUC = 0.79, 95% CI 0.68–0.87 *P* < 0.001), comparing with Model 1 (AUC = 0.87, 95% CI 0.76–0.92 *P* < 0.001) (*P* = 0.049).

## Discussion

ApoA-IV is a cardiovascular protective factor and exerts anti-inflammatory and anti-oxidative stress effects [11–16]. The present study has demonstrated that serum apoA-IV levels are significantly associated with FMD in T2DM patients. Serum apoA-IV levels are higher in patients with high FMD. In logistic analysis, decreased serum apoA-IV level was an independent determinant of low FMD in T2DM patients. In linear regression analysis, apoA-IV was independently correlated with FMD. Our findings have suggested a notion that apoA-IV protects endothelial function as represented by FMD in diabetic patients, consistent with previous evidence [11–16].

It has been evidenced that the endothelial function is the main element of vascular homeostasis regarding vasoconstriction-vasodilation regulation, anti-inflammatory and anticoagulant properties. Among endothelium-derived mediators, nitric oxide suppresses cell inflammation and inflammatory cell adhesion, inhibits thrombosis, facilitates blood flow, and limits vessel wall remodeling [20]. Endothelial dysfunction is a diffuse vascular disorder characterized by reduced NO bioavailability. It occurs at early stage of atherosclerosis, and progresses throughout the whole atherosclerosis process, which significantly aggravates under diabetic condition [21]. FMD of peripheral conduit arteries is one of the common tests for endothelial function. Previous study shows that FMD is lower in T2DM group versus control group [22]. FMD surveillance may have prognostic significance. Since FMD responds rapidly to treatment, it is used to verify drug efficacy and to evaluate bioactive substances [23].

In the present study, serum apoA-IV levels were positively associated with FMD in T2DM patients and decreased apoA-IV level was an independent determinant of low FMD in diabetic patients in logistic regression analysis. Our results suggest that apoA-IV protects endothelial function and subsequently prevent atherogenesis, which is consistent with previous studies [11–16]. Previous researches have also demonstrated that in animal models, apoA-IV transgenic mice reveal remarkable attenuation in atherogenesis after western diet as compared with control mice [24]. ApoE-/- mice with overexpression or infusion of human apoA-IV manifest less atherosclerotic lesions with steady fiber cap and smaller lipid core [16, 25, 26]. In human, serum apoA-IV concentrations negatively correlate with CAD in Caucasian, Asian Indian and Chinese population [27, 28] and also with chronic kidney disease in a prospective cohort study [29]. Our findings add novel information regarding apoA-IV function, jointly supporting apoA-IV as a cardiovascular protective factor.

### Limitations of the study

First, the study is a cross-sectional study, aiming to investigate the relationship between FMD and apoA-IV, but not causative links. Second, apoA-IV has various modification forms in diabetic patients, which may influence the biological functions of apoA-IV [17]. In our future studies, prospective study regarding the relation of endothelial function and apoA-IV level or modifications of apoA-IV will be done.

## Conclusion

Serum apoA-IV levels are associated with FMD in patients with T2DM, suggesting that apoA-IV protects endothelial function in patients with T2DM.

## Data Availability

The datasets generated and/or analysed during the current study are not publicly available due to patients’ privacy protection, but are available from the corresponding author on reasonable request.
